## [Reviewer comments · Life Science Alliance]

Unique activities of two overlapping PAX6 retinal enhancers

Kirsty Uttley, Andrew Papanastasiou, Manuela Lahne, Jennifer Brisbane, Ryan Macdonald, Wendy Bickmore, and Shipra Bhatia
DOI: <https://doi.org/10.26508/lsa.202302126>

Corresponding author(s): Shipra Bhatia, MRC Human Genetics Unit, MRC IGMM, University of Edinburgh

Review Timeline:	Submission Date:	2023-05-01
	Editorial Decision:	2023-05-03
	Revision Received:	2023-07-25
	Editorial Decision:	2023-08-08
	Revision Received:	2023-08-16
	Accepted:	2023-08-17

Transaction Report:

Please note that the manuscript was reviewed at Review Commons and these reports were taken into account in the decision-making process at *Life Science Alliance*

Reviews

Review #1

****Summary:****

In the article entitled "Unique functions of two overlapping PAX6 retinal enhancers", Uttley and coworkers characterize in detail the activity of two conserved human enhancers (i.e. NRE and HS5) previously reported to drive Pax6 expression to the neural retina. By integrating these enhancers in a PhiC31 landing site using a dual enhancer-reporter cassette, they generated a zebrafish stable line in which their activity can be followed by the expression of GFP (NRE) and mCherry (HS5). The authors show that although the enhancers have a partially overlapping activity at early stages (24hpf), later on (48 and 72hpf) they activity domains segregate: to stem cells and differentiated amacrine cells for NRE, and to proliferating progenitors and differentiated Müller glia cells for HS5. To this end they used two different approaches: a scRNA-seq analysis of sorted cells from the transgenic line and a immunofluorescent analysis employing cell specific markers. The authors conclude that their analysis allowed the identification of unique cell type-specific functions.

****Major comments:****

In general terms, the article is technically sound (please, see section B for an assessment of the significance of the findings). The methodology used and the data analysis are accurate. The work is well presented, the figures are clear, and the previous literature properly cited. My main concerns are the following:

1. A general concern on the main conclusion of the work "the identification of unique cell type-specific functions for these enhancers". This is in my opinion only partially addressed by the study, as the conclusions are limited due to the absence of genetic experiments: such as deleting the enhancers in their native genomic context (either in human organoids or the homologous sequence in animal models), or at least assessing the effect of mutating their sequence in transgenesis assays in zebrafish. I understand that these functional assays may be out of the scope of the current work, but then the text should be toned down (the word "function" is extensively used) to make clear that the authors mean just expression. I would suggest substituting the word by "activity" in many instances.

The absence of further genetic experiments also limits the significance of the study (see section B).

2. Whereas the work in general is technically correct (particularly transgenic lines and scRNA-seq data are well described and presented), the co-expression analyses using cell-specific markers (figure 5) need to be improved. There are several issues here. First, the magnification shown is too low to appreciate the colocalization details in the figure. The panels should be replaced by others with higher magnification/resolution (see also minor comment on color-blind compatible images)

In addition, the selection of the markers is suboptimal. Although PCNA is a good general marker of the entire CMZ, it would be advisable to repeat the experiments using more specific markers of the stem cell niche (e.g. rx1, vsx2; Raymond et al 2006; BMC dev Biol) to better define the enhancers expression domain. In addition, HuC/D labels both RGCs and amacrine, and the colocalization could also be refined using amacrine specific markers (e.g. ptf1a : Jusuf & Harris 2009, Neural Dev).

****Minor comments:****

1. The work includes several figures (1, 2, 5, 6 and S1) showing colocalization experiments in

which channels are shown in red and green. I would advise replacing the red channel with magenta (or the green with cyan) in order to make the figures accessible to readers with color-blindness. This also applies to the schematic representations in figure 6.

2. It is unclear in the text/images whether the expression driven by the HS5 enhancer is exclusively restricted to temporal retina throughout development (By the way, this differential nasal vs temporal expression should also be included in the final scheme in Figure 6). Does this mean that the expression of Pax6 in proliferating progenitors and Müller glia cells in the nasal retina is not controlled by this enhancer? To which extent is Pax6 needed to maintain the identity of these cell types?

3. The following sentence in the Discussion "To the best of our knowledge, ours is the first report where the activities of developmental enhancers have been mapped in vivo at single-cell resolution to reveal distinct patterns of activity" should be removed/rephrased. I would argue that the activity of cis-regulatory regions associated to any developmental gene are genome-wide mapped at single cell resolution in each scATACseq experiment.

4. In the methods section:

- (a) FACS experiments: Please provide a supplementary Figure to graphically account for all gating/sorting strategies.

- (b) ScRNA-seq analysis: Please provide the values of mean reads per cell and median genes per cell as obtained from Cell Ranger. This would be informative for others performing similar experiments

****Referees cross-commenting****

I agree with the comments by reviewer #2 on the FACsorting experiments, the description of the landing sites, and the limited significance of the results.

As described in the previous section, the technical quality of this work is high in general terms. The experiments presented are clear and the conclusions straightforward. In that sense, the study will be a useful reference for those interested in the regulatory logic of Pax6 during eye development, including mainly developmental biologists and human geneticists. This may be particularly the case if new variants can be associated with these enhancers in microphthalmic patients.

The significance and novelty of the findings is however limited by several factors:

- a) First, although the level of detail described in this article was not achieved previously, the human enhancers NRE and HS5 (or their conserved homologous in other vertebrates) were previously reported to drive Pax6 expression to the neural retina in transgenesis assays.(Kammandel et al 1999; Marquardt et al 2001; McBride et al 2011; Ravi et al 2013; Kim et al 2017).

- b) As mentioned in the previous section, the transgenesis assays are not complemented with genetic experiments. The function of the enhancers on retina differentiation and cell fate determination could have been investigated either by deleting them (or their homologous in different species) in their native context, or by exploring their regulatory grammar introducing point mutations or micro-deletions in transgenesis assays.

- c) For reasons not explained in the text, the analysis focuses only in two of the many cis-regulatory regions controlling Pax6 expression in the retina (Lima Cunha et al 2019, Genes). In the absence of a more comprehensive analysis is difficult assessing the relevance of the findings here described.

- d) Finally, from a very general methodological point of view, the approach of using scRNA-seq to investigate enhance activity at a single-cell level is valid and original. However, it is unclear to which extent will be a useful method for many studies, particularly if the activity of endogenous

elements is being assessed. In such cases, available scATAC-seq data will provide genome-wide information on the activity of any cis-regulatory element with cell resolution with no need for transgenesis assays and sorting experiments.

Review # 2

In this work, Uttley et al fine characterize two previously described Pax6 retinal enhancers (NRE and HS5) by combining QSTARZ transgenesis method in zebrafish (allowing to produce site-specific integrations of a dual enhancer reporter cassette), scRNAseq and co-immunostaining with specific markers for different retinal cell populations.

The work is experimentally very well performed and well presented and only minor considerations are raised below:

- Authors observe that a large fraction Of FACs sorted cells do not display expression of mCherry or EGFP RNAm in their scRNAseq analysis and attribute this to read dropout in the scRNAseq data and/ or to false-positive FAC cell selection. However, a third possibility exists: n fact due to the high stability of the EGFP and mCherry reporters cells or their progeny could maintain relatively high levels of these reporters even after transcriptional downregulation. Accordingly, the two reporters are strongly expressed in retinal precursor at early stages (24hpf). Thus, in my opinion, it is possible that some cells expressing these reporters retained significant EGFP/mCherry protein levels at 48hpf. Could the authors comment on this? Besides, authors could provide the FACsorting data to give an idea of whether only highly EGFP/ mCherry expressing cells were selected or whether also the low or mild expressing ones were included in the scRNAseq analysis. Finally, a combination of HCR/FSH and GFP//mCherry immunostaining could be used to assess whether a discrepancy in the protein vs mRNA distribution of the reporters exists.
- The authors could provide the information on the landing site used for the QSTARZ transgene integration. While from their previous publication (Bhatia et al 2021) I assume it is the chr6 landing site, it would be worth having this information in the manuscript, as well as a genotyping validation of the correct integration.

Referees cross-commenting

I agree with all the points raised by reviewer 1. Particularly I also find that scATACseq experiments already allow testing, to some extent, enhancer activity at cellular level.

From the biological point of view the work provides only an incremental advance in our understanding of the functions of the HS5 and NRE PAX6 enhancers and of PAX6 regulation in the retina. In fact, unraveling the precise contribution of these enhancers to Pax6 retinal expression and the trans-regulatory code controlling their activity would require complex genetic experiments and would fall out of the scope of this work, requiring an extensive amount of work which could not be addressed in the short term. Thus, this work should be regarded as a methodological resource, with its main strength consisting of the use scRNAseq to fine-characterize enhancer activity.

May 3, 2023

Re: Life Science Alliance manuscript #LSA-2023-02126

Dr. Shipra Bhatia
MRC Human Genetics Unit, MRC IGMM, University of Edinburgh
Western General Hospital
Crewe Road South
Edinburgh EH4 2XU
United Kingdom

Dear Dr. Bhatia,

Thank you for submitting your manuscript entitled "Unique functions of two overlapping PAX6 retinal enhancers" to Life Science Alliance. We invite you to re-submit the manuscript, revised according to your Revision Plan.

Thank you for this interesting contribution to Life Science Alliance. We are looking forward to receiving your revised manuscript.

Sincerely,

B. MANUSCRIPT ORGANIZATION AND FORMATTING:

Corresponding author(s): Shipra Bhatia

1. General Statements [optional]

Our study aims to uncover precise cell-type specific activities of development enhancers using a combination of high-resolution live imaging and single-cell RNA sequencing. Defining the precise cell-types where the enhancers drive expression of their target genes is a vital step towards understanding the functional relevance of disease-associated sequence variation in the human genome. We believe the methods we describe here would be widely employed to understand the activities of developmental enhancers with mutations associated with human diseases. Both the reviewers have acknowledged the technical strength of the methods we describe in this manuscript.

2. Description of the planned revisions

We thank the reviewers for their enthusiastic support for our work and their insightful comments and suggestions which we believe strengthen the manuscript. Below we detail how we have responded to each of the specific points raised by each reviewer in the revised version of our manuscript.

Reviewer #1 (Evidence, reproducibility and clarity (Required)):

Summary:

In the article entitled "Unique functions of two overlapping PAX6 retinal enhancers", Uttley and coworkers characterize in detail the activity of two conserved human enhancers (i.e. NRE and HS5) previously reported to drive Pax6 expression to the neural retina. By integrating these enhancers in a PhiC31 landing site using a dual enhancer-reporter cassette, they generated a zebrafish stable line in which their activity can be followed by the expression of GFP (NRE) and mCherry (HS5). The authors show that although the enhancers have a partially overlapping activity at early stages (24hpf), later on (48 and 72hpf) they activity domains segregate: to stem cells and differentiated amacrine cells for NRE, and to proliferating progenitors and differentiated Müller glia cells for HS5. To this end they used two different approaches: a scRNA-seq analysis of sorted cells from the transgenic line and a immunofluorescent analysis employing cell specific markers. The authors conclude that their analysis allowed the identification of unique cell type-specific functions.

Major comments:

In general terms, the article is technically sound (please, see section B for an assessment of the significance of the findings). The methodology used and the data analysis are accurate. The work is well presented, the figures are clear, and the previous literature properly cited. My main concerns are the following:

1) A general concern on the main conclusion of the work "the identification of unique cell type-specific functions for these enhancers". This is in my opinion only partially addressed by the study, as the conclusions are limited due to the absence of genetic experiments: such as deleting the enhancers in their native genomic context (either in human organoids or the homologous sequence in animal models), or at least assessing the effect of mutating their sequence in transgenesis assays in zebrafish. I understand that these functional assays may be out of the scope of the current work, but then the text should be toned down (the word "function" is extensively used) to make clear that the authors mean just expression. I would suggest substituting the word by "activity" in many instances.

The absence of further genetic experiments also limits the significance of the study (see section B).
We appreciate and agree with the reviewer's concern. We have now substituted the word "function" with "activity" throughout the manuscript, including the title

2) Whereas the work in general is technically correct (particularly transgenic lines and scRNA-seq data are well described and presented), the co-expression analyses using cell-specific markers (figure 5) need to be improved. There are several issues here.

First, the magnification shown is too low to appreciate the colocalization details in the figure. The panels should be replaced by others with higher magnification/resolution (see also minor comment on color-blind compatible images)

High-magnification panels are now included in Fig 5 and Fig S7.

In addition, the selection of the markers is suboptimal. Although PCNA is a good general marker of the entire CMZ, it would be advisable to repeat the experiments using more specific markers of the stem cell niche (e.g. rx1, vsx2; Raymond et al 2006; BMC dev Biol) to better define the enhancers expression domain. In addition, HuC/D labels both RGCs and amacrine, and the colocalization could also be refined using amacrine specific markers (e.g. ptf1a : Jusuf & Harris 2009, Neural Dev).

We appreciate the reviewer's comments. We would like to clarify that we were limited by the choice of markers for immunohistochemistry due to the availability of antibodies. Since our constructs contain eGFP and mCherry for tracking enhancer activity, we were also unable to use the transgenic lines available for additional marker genes for co-localisation experiments. Any cell that is HuC/D positive in the inner nuclear layer is an amacrine cell and we have now also included parvalbumin as an additional marker for amacrine cells in the INL (Fig S7).

Minor comments:

1.- The work includes several figures (1, 2, 5, 6 and S1) showing colocalization experiments in which channels are shown in red and green. I would advise replacing the red channel with magenta (or the green with cyan) in order to make the figures accessible to readers with color-blindness. This also applies to the schematic representations in figure 6.

We have changed the channel colours throughout the manuscript as suggested by the reviewer

2.- It is unclear in the text/images whether the expression driven by the HS5 enhancer is exclusively restricted to temporal retina throughout development (By the way, this differential nasal vs temporal expression should also be included in the final scheme in Figure 6).

Fig 6 is now modified accordingly. We only detect activity of HS5 up to ~72hpf (supplementary movie 1) and only in the temporal retina.

Does this mean that the expression of Pax6 in proliferating progenitors and Müller glia cells in the nasal retina is not controlled by this enhancer?

We would predict this based on our results. However this can only be said conclusively once HS5 is deleted and changes in PAX6 expression are assessed.

To which extent is Pax6 needed to maintain the identity of these cell types?

This is described in detail in the discussion section (290-300). The activity of PAX6 in these cells do not appear to be restricted to the nasal retina, but it could be regulated by another retinal enhancer in the locus.

3.- The following sentence in the Discussion "To the best of our knowledge, ours is the first report where the activities of developmental enhancers have been mapped in vivo at single-cell resolution to reveal distinct patterns of activity" should be removed/rephrased. I would argue that the activity of cis-regulatory regions associated to any developmental gene are genome-wide mapped at single cell resolution in each scATACseq experiment.

We agree that scATAC-seq gives information about potentially active enhancers but it does not define the precise cell-types unless overlapped with expression data. Our method is aimed at 'defining' the precise cell-types where the enhancer is active and has the potential to be used to build high resolution maps of cell-type specific enhancer usage for loci with multiple enhancers driving a single gene. We have rephrased the sentence in the discussion to emphasise this (lines 333-336). We have also added details of activities of NRE and HS5 in published scATAC-seq data from adult human retinae, which support our conclusions on the cell-type specific activities of these enhancers (line 277-279, 292-294). We have also modified the text in the discussion to emphasise the power of our method (lines 316-351).

4.- In the methods section:

(a) FACS experiments: Please provide a supplementary Figure to graphically account for all gating/sorting strategies.

This is included in the revised manuscript as Fig S2

(b) ScRNA-seq analysis: Please provide the values of mean reads per cell and median genes per cell as obtained from Cell Ranger. This would be informative for others performing similar experiments

This information is now included in the materials and methods section (lines 472-473).

****Referees cross-commenting****

I agree with the comments by reviewer #2 on the FACSorting experiments, the description of the landing sites, and the limited significance of the results.

Please see our response to those comments below.

Reviewer #1 (Significance (Required)):

As described in the previous section, the technical quality of this work is high in general terms. The experiments presented are clear and the conclusions straightforward. In that sense, the study will be

a useful reference for those interested in the regulatory logic of Pax6 during eye development, including mainly developmental biologists and human geneticists. This may be particularly the case if new variants can be associated with these enhancers in microphthalmic patients.

The significance and novelty of the findings is however limited by several factors:

a) First, although the level of detail described in this article was not achieved previously, the human enhancers NRE and HS5 (or their conserved homologous in other vertebrates) were previously reported to drive Pax6 expression to the neural retina in transgenesis assays. (Kammandel et al 1999; Marquardt et al 2001; McBride et al 2011; Ravi et al 2013; Kim et al 2017).

We agree that the enhancers we describe in this study have been studied before. However, we would like to argue that ours is the first study where we define precise cell-types for the activity of these enhancers, revealing potentially non-redundant functions. We have revised the discussion to strengthen this argument (lines 316-351).

b) As mentioned in the previous section, the transgenesis assays are not complemented with genetic experiments. The function of the enhancers on retina differentiation and cell fate determination could have been investigated either by deleting them (or their homologous in different species) in their native context, or by exploring their regulatory grammar introducing point mutations or micro-deletions in transgenesis assays.

We agree that the suggested experiments would be useful for unambiguously establishing the functions of these enhancers and these will be the focus of the future studies in our lab or others interested on PAX6-mediated gene regulation. We have discussed these prospects in the revised version of the manuscript (lines 348-351).

c) For reasons not explained in the text, the analysis focuses only in two of the many cis-regulatory regions controlling Pax6 expression in the retina (Lima Cunha et al 2019, Genes). In the absence of a more comprehensive analysis is difficult assessing the relevance of the findings here described.

NRE and HS5 enhancers were chosen for this analysis as previous work had demonstrated potentially overlapping domains and stages of activities for these enhancers (described in lines 107-116). We thus considered them good candidates for further analysis to investigate if distinct or overlapping cell-type specific activities could be uncovered in our assays. We agree that other enhancers for the PAX6 locus should be investigated using similar analysis pipeline to build a complete picture of the enhancer mediated regulation of PAX6. This has been added to the discussion (lines 348-351).

d) Finally, from a very general methodological point of view, the approach of using scRNA-seq to investigate enhance activity at a single-cell level is valid and original. However, it is unclear to which extent will be a useful method for many studies, particularly if the activity of endogenous elements is being assessed. In such cases, available scATAC-seq data will provide genome-wide information on the activity of any cis-regulatory element with cell resolution with no need for transgenesis assays and sorting experiments..

We thank the reviewer for recognising the novelty of the approach we describe in this manuscript. We have discussed the merits and demerits of our method in the revised version of the manuscript (lines 316-351).

Reviewer #2 (Evidence, reproducibility and clarity (Required)):

In this work, Uttley et al fine characterize two previously described Pax6 retinal enhancers (NRE and HS5) by combining QSTARZ transgenesis method in zebrafish (allowing to produce site-specific integrations of a dual enhancer reporter cassette), scRNAseq and co-immunostaining with specific markers for different retinal cell populations.

The work is experimentally very well performed and well presented and only minor considerations are raised below:

- Authors observe that a large fraction Of FACs sorted cells do not display expression of mCherry or EGFP RNAm in their scRNAseq analysis and attribute this to read dropout in the scRNAseq data and/ or to false-positive FAC cell selection. However, a third possibility exists: n fact due to the high stability of the EGFP and mCherry reporters cells or their progeny could maintain relatively high levels of these reporters even after transcriptional downregulation. Accordingly, the two reporters are strongly expressed in retinal precursor at early stages (24hpf). Thus, in my opinion, it is possible that some cells expressing these reporters retained significant EGFP/mCherry protein levels at 48hpf. Could the authors comment on this? Besides, authors could provide the FACsorting data to give an idea of whether only highly EGFP/ mCherry expressing cells were selected or whether also the low or mild expressing ones were included in the scRNAseq analysis. Finally, a combination of HCR/FSH and GFP//mCherry immunostaining could be used to assess whether a discrepancy in the protein vs mRNA distribution of the reporters exists.

We agree with the reviewer's concerns and are working on a future iteration of our assay using de-stabilized fluorophores (eg. Ds-red) to make the analysis pipeline bias-free.

The requested FACS data is provided in the revised version of the manuscript (Fig S2).

- The authors could provide the information on the landing site used for the QSTARZ transgene integration. While from their previous publication (Bhatia et al 2021) I assume it is the chr6 landing site, it would be worth having this information in the manuscript, as well as a genotyping validation of the correct integration.

We have added an elaborate description of how we select transgenic lines in the materials and methods section (lines 374-376).

****Referees cross-commenting****

I agree with all the points raised by reviewer 1. Particularly I also find that scATACseq experiments already allow testing, to some extent, enhancer activity at cellular level.

Reviewer #2 (Significance (Required)):

From the biological point of view the work provides only an incremental advance in our

understanding of the functions of the HS5 and NRE PAX6 enhancers and of PAX6 regulation in the retina. In fact, unraveling the precise contribution of these enhancers to Pax6 retinal expression and the trans-regulatory code controlling their activity would require complex genetic experiments and would fall out of the scope of this work, requiring an extensive amount of work which could not be addressed in the short term. Thus, this work should be regarded as a methodological resource, with its main strength consisting of the use scRNAseq to fine-characterize enhancer activity.

3. Description of the revisions that have already been incorporated in the transferred manuscript

None

4. Description of analyses that authors prefer not to carry out

None

August 8, 2023

RE: Life Science Alliance Manuscript #LSA-2023-02126R

Dr. Shipra Bhatia
MRC Human Genetics Unit
University of Edinburgh
Western General Hospital
Crewe Road South
Edinburgh EH4 2XU
United Kingdom

Dear Dr. Bhatia,

Thank you for submitting your revised manuscript entitled "Unique activities of two overlapping PAX6 retinal enhancers". We would be happy to publish your paper in Life Science Alliance pending final revisions necessary to meet our formatting guidelines.

- please address Reviewer 1's remaining minor comment
- please add your main, supplementary figure, and table legends as separate legends to the main manuscript text after the references section
- please use the [10 author names, et al.] format in your references (i.e. limit the author names to the first 10)
- please add a callout for Fig SA, Fig SB, Fig SC, Fig S7A, Fig S7B to your main manuscript text
- please update your Data Availability statement with the GEO accession information and the GitHub link for the code

A. FINAL FILES:

B. MANUSCRIPT ORGANIZATION AND FORMATTING:

Sincerely,

Reviewer #1 (Comments to the Authors (Required)):

This is a revised version of the manuscript "Unique activities of two overlapping PAX6 retinal enhancers" by Uttley et al.

I had previous concerns on the significance of the study as well as a few technical comments. However, provided that functional experiments (to evaluate the function of the Pax6 enhancers) are likely out of the scope of this journal, which main criterion is that the data support the claims made, my main criticisms have been addressed properly in this revised version.

In particular: (1) High magnification panels, provided with the new set of color-blind-friendly figures, clearly sustain the authors' conclusions. (2) Additional methodological details have been included as requested, and more importantly (3) claims on the functional relevance of the findings have been toned down.

I only have a very minor additional (optional) comment: It would be nice to comment in the discussion section on the prediction that the HS5 enhancer is not controlling Pax6 expression in progenitors and Muller cells at the nasal retina.

Reviewer #1

This is a revised version of the manuscript "Unique activities of two overlapping PAX6 retinal enhancers" by Uttley et al.

I had previous concerns on the significance of the study as well as a few technical comments. However, provided that functional experiments (to evaluate the function of the Pax6 enhancers) are likely out of the scope of this journal, which main criterion is that the data support the claims made, my main criticisms have been addressed properly in this revised version.

In particular: (1) High magnification panels, provided with the new set of color-blind-friendly figures, clearly sustain the authors' conclusions. (2) Additional methodological details have been included as requested, and more importantly (3) claims on the functional relevance of the findings have been toned down.

I only have a very minor additional (optional) comment: It would be nice to comment in the discussion section on the prediction that the HS5 enhancer is not controlling Pax6 expression in progenitors and Muller cells at the nasal retina.

We have modified the discussion to include the information requested by the reviewer (lines 303-318 of the revised manuscript).

August 17, 2023

RE: Life Science Alliance Manuscript #LSA-2023-02126RR

Dr. Shipra Bhatia
MRC Human Genetics Unit, MRC IGMM, University of Edinburgh
Western General Hospital
Crewe Road South
Edinburgh EH4 2XU
United Kingdom

Dear Dr. Bhatia,

Thank you for submitting your Research Article entitled "Unique activities of two overlapping PAX6 retinal enhancers". It is a pleasure to let you know that your manuscript is now accepted for publication in Life Science Alliance. Congratulations on this interesting work.

DISTRIBUTION OF MATERIALS:

Again, congratulations on a very nice paper. I hope you found the review process to be constructive and are pleased with how the manuscript was handled editorially. We look forward to future exciting submissions from your lab.

Sincerely,
